# Toluene-Containing Gas Stream Treatment by Persulfate-Based Oxidation: Process Variables Affecting Mass Transfer

**DOI:** 10.1021/acs.iecr.5c00924

**Published:** 2025-08-29

**Authors:** Ana S. P. Alves, Carmen S. D. Rodrigues, João M. Miranda, Luís M. Madeira

**Affiliations:** 1 LEPABE − Laboratory for Process Engineering, Environment, Biotechnology and Energy, Faculty of Engineering, 112048 University of Porto, Rua Dr. Roberto Frias, Porto 4200-465, Portugal; 2 ALiCE − Associate Laboratory in Chemical Engineering, Faculty of Engineering, University of Porto, Rua Dr. Roberto Frias, Porto 4200-465, Portugal; 3 CEFT − Transport Phenomena Research Center, Faculty of Engineering, University of Porto, Rua Dr. Roberto Frias, Porto 4200-465, Portugal

## Abstract

A toluene-containing gas stream was treated through the application of an activated persulfate-based advanced oxidation process (AOP). As in this type of AOP pollutant degradation is carried out in the liquid phase, the gas-to-liquid mass transfer process is a crucial step to which great attention should be paid, this being the main goal of the present study. A careful analysis of the impact of some parameters on toluene’s degradation was performed, investigating (i) the effect of reactor configuration (bubble reactor *vs*. bubble column reactor with different aspect ratios), (ii) the influence of the type of diffuser (aquarium diffusers, with different geometric shapes, *vs.* diffuser plates, with different porosities, i.e., with different pore sizes), and (iii) the impact of the water matrix (distilled water *vs.* tap water). The best treatment performance was achieved when the bubble column reactor and the diffuser plate with higher porosity were used, being indifferent to the type of liquid phase selected, which, from the practical point of view, brings important advantages as tap water can be used without affecting process performance.

## Graphical abstract

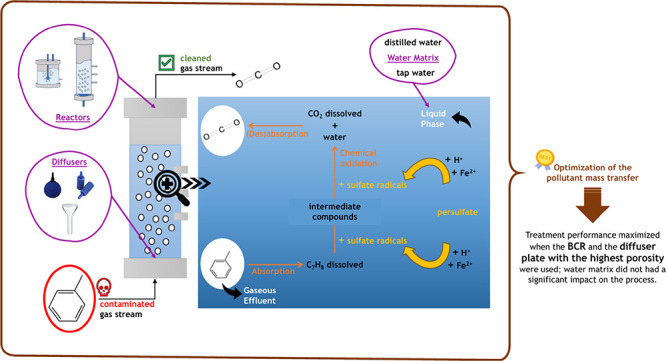

## 1 Introduction

There are several types of volatile organic compounds (VOCs) that are emitted into the atmosphere every day. VOCs are volatile and photochemically reactive species, which exhibit physical and chemical properties that allow them a great capacity for dispersion.^1,2^ Thus, once released into the environment, they can volatilize, dissolve in water, and/or adhere to soil particles, making it impossible to control the resulting environmental and health repercussions.^1,2^

Toluene is one of the most common VOCs found in industrial gas effluents derived from chemical and petrochemical industries; it is a component of gasoline and has been commonly used as a solvent for industrial purposes.^3^ However, its emission to the environment must be avoided, since according to the US Environmental Protection Agency (US EPA), toluene is a toxic and carcinogenic compound for both humans and animals, apart from being harmful to the environment.^3–6^

There are already documented in the literature some treatment processes that have been applied to decontaminate toluene-containing gaseous streams, among which are catalytic ozonation,^7^ adsorption,^8^ Fenton^9,10^ and photo-Fenton’s reaction,^11^ biofiltration,^12^ and dielectric barrier discharge combined with distinct catalysts;^13^ however, all of them present some disadvantages (please refer to Section A of the Supporting Information). Recently, some works have been published where toluene-containing gas streams are treated through the application of activated persulfate-based advanced oxidation processes (activated PS-based AOPs), with quite encouraging results, as summarized in Section B of the Supporting Information.

Activated PS-based AOPs have shown to be promising in terms of treating priority pollutants, as they allow the real elimination of toxic and non-biodegradable compounds in an efficient, fast, and not expensive way.^14,15^ Still, they make use of environmental-friendly reagents and mild conditions of temperature and pressure,^16^ apart from not producing secondary pollution.^16^ Basically, in this type of physical–chemical processes occurs the *in-situ* generation of active species (particularly sulfate radicals, SO_4_^·–^) which, due to their high oxidation power (*E*_0_ = 2.60 eV), react with the organic pollutant (OP), degrading it.^17–19^ It should nevertheless be mentioned that in this type of treatment processes, apart from the formation of sulfate radicals, there are some other reactive oxygen species that can also be present in the treatment system, like hydroxyl radicals (HO^·^), superoxide anion radicals (O_2_^·–^), or even singlet oxygen (^1^O_2_); however, as previously reported by the authors,^20^ under the operating conditions used in the present study, the amount of hydroxyl radicals available to perform toluene’s degradation is low/not significant when compared to the sulfate radicals, while superoxide anions and singlet oxygen are more likely to be formed when the treatment system is working under alkaline conditions. Obviously, depending on the target pollutant OP, it can be partially degraded to intermediate compounds (OP′, eq 1), which are usually less toxic and that can then be treated using biological processes;^21,22^ hopefully, such intermediates can be completely oxidized by the sulfate radicals into harmless components such as carbon dioxide (CO_2_) and water (H_2_O) (eq 2):^22^

$$\mathrm{OP}+{\mathrm{SO}}_{4}^{\cdot -}\to {\mathrm{OP}}^{’}$$ 1

$${\mathrm{OP}}^{’}+{\mathrm{SO}}_{4}^{\cdot -}\to {\mathrm{CO}}_{2}+H_{2}O$$ 2

Sulfate radicals are formed through the activation of persulfate (PS; S_2_O_8_^2–^). PS has been playing an important role as oxidizing agent, with some studies calling it an emerging oxidant, given the excellent results that have been obtained in this area.^18,23^ However, as PS is a very stable compound, it will react slowly with the organic pollutants; nonetheless, this can be overcome with its previous activation.^24^ This action can be performed by the application of several methods like heat, radiation, ultrasounds, transition metal ions/catalyst, and/or pH change,^22^ leading to the formation of free sulfate radicals, which are stronger from the oxidative point of view, making the degradative process more efficient.^19,25–27^ In this study, PS will be activated through the use of transition metal ions/catalyst, namely, Fe^2+^ (eq 3)^28,29^ and pH variation, under acidic conditions (eq 4):^30^

$$S_{2}O_{8}^{2-}+{\mathrm{Fe}}^{2+}\to {\mathrm{Fe}}^{3+}+{\mathrm{SO}}_{4}^{\cdot -}+{\mathrm{SO}}_{4}^{2-}$$ 3

$$S_{2}O_{8}^{2-}+H^{+}\to HS_{2}O_{8}^{-}\to {\mathrm{SO}}_{4}^{2-}+{\mathrm{SO}}_{4}^{\cdot -}+H^{+}$$ 4

In this AOP, the contaminated gas stream is treated through a liquid-phase oxidation, making it necessary to use a bubble reactor (BR) or a bubble column reactor (BCR). For that to happen, the contaminated gas stream needs to be continuously dispersed within the water, present inside the reactor, promoting the contact needed between the target pollutant and the oxidizing agent generated therein (both PS and iron ions are previously dissolved in the water). With this contact, the gaseous pollutant is transferred to the liquid phase, through the bubbling, by absorption; once dissolved in the liquid phase, the pollutant is degraded/oxidized, due to the presence of the sulfate radicals.^9,31^ This oxidation will lower the concentration of the target VOC within the liquid phase, increasing the driving force for the transfer of more pollutant from the gas stream to the water.^9,31^ Figure 1 shows a scheme of the treatment process employed.

**Figure 1:**
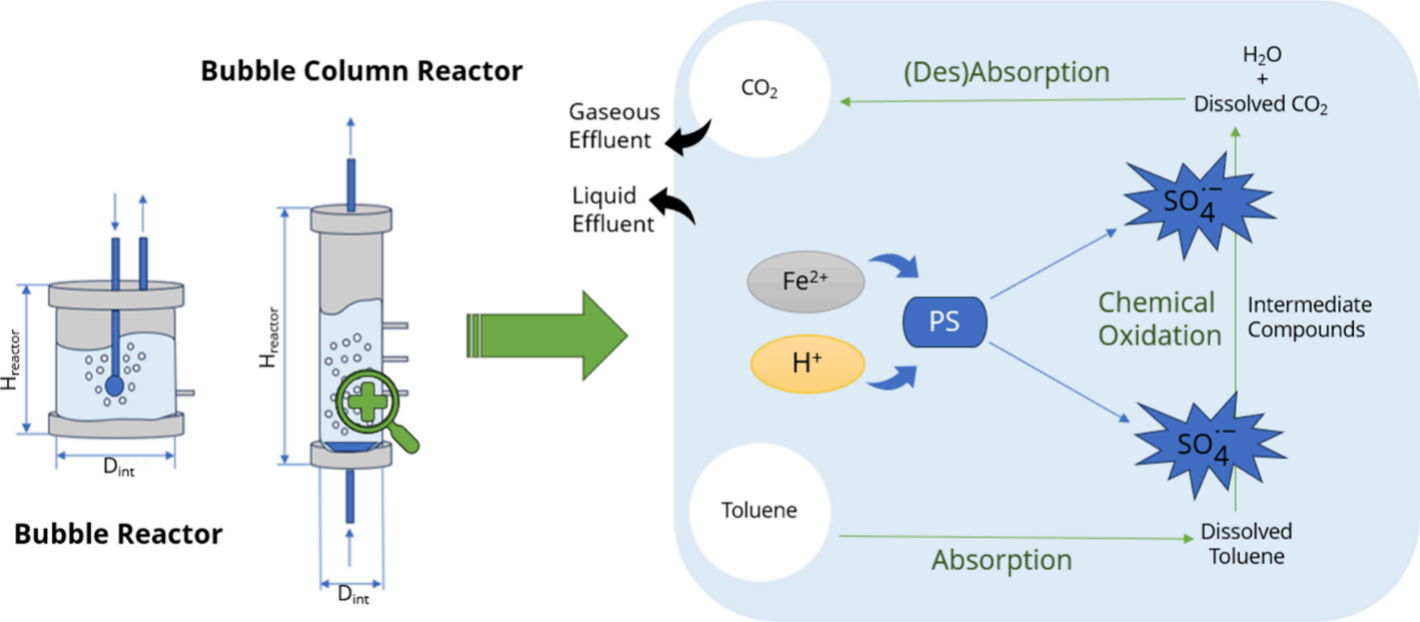
Scheme of the degradative process of toluene using an activated persulfate-based AOP in the liquid phase.

In this study, it is intended for the first time to analyze and better understand the impact that some parameters of the reactor itself have on the mass transfer and, consequently, on the degradation of the model pollutant (by accounting for the amount of toluene transferred/oxidized). This will be done by selecting the most suitable reactor’s configuration aspect ratio, optimizing the type of diffuser, and analyzing the effect of the bubbles’ size. Additionally, the effect of the water matrix used (distilled *vs*. tap water) will be also investigated.

## 2 Material and Methods

### 2.1 Chemical Reagents

The model pollutant, toluene (C_7_H_8_; 100% purity), was acquired from VWR Chemicals. The oxidant agent, potassium peroxydisulfate (K_2_S_2_O_8_; 99%), was obtained from Alfa Aesar. The catalyst, iron (II) sulfate heptahydrate (FeSO_4_·7H_2_O; 99%), was purchased from Panreac. For pH adjustment, sulfuric acid (1 M H_2_SO_4_; 96.4%) was used, which was acquired from VWR Chemicals. For PS quantification, ammonium iron (II) sulfate hexahydrate (or ferrous ammonium sulfate —FAS, (NH_4_)_2_Fe­(SO_4_)_2_·6H_2_O; 99%) and ammonium thiocyanate (NH_4_SCN; 99.7%) were used, both obtained from VWR Chemicals. Distilled water was used to prepare all of the solutions.

### 2.2 Experimental Setup and PS-Based AOP Runs

Although the present study was carried out in two reactors—a bubble reactor and a bubble column reactor (information regarding the reactors is presented in Table 1)— the experimental facility used was the same (scheme of the experimental facility shown in Figure 2), being only the reactor replaced. Both reactors were cylindrical acrylic tubes with an inlet and an outlet port for the gaseous effluent that was continuously bubbled within the water present inside the reactor (the treatment system was configured to operate in a semi-continuous mode, i.e., the liquid phase was fed discontinuously and the gaseous stream was fed continuously); the gas effluent entered the reactor and was dispersed through a diffuser, and both reactors had liquid sampling points, which were on the bottom of the reactor (in the BR) or along the reactor, at different heights (in the BCR)—*cf*. Figure 2.

**Table 1:** Physical Dimensions of the Reactors Used: Bubble Reactor (BR) and Bubble Column Reactor (BCR)

Type of reactor	BR	BCR
*H*_reactor_ (m)	0.130	1.000
*D*_int_ (m)	0.090	0.025
*H*_liquid_ (m)	0.047	0.610
*H*_liquid_/*D*_int_	0.52	24.4

**Figure 2:**
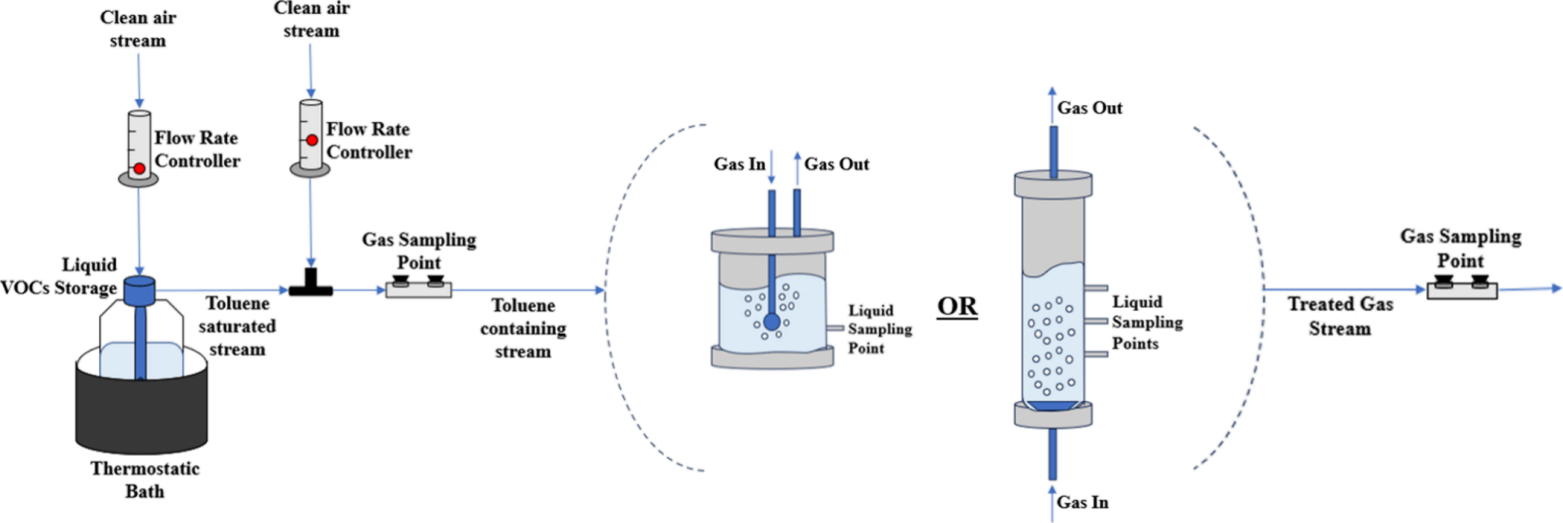
Schematics of the experimental set-up employed for the persulfate-based oxidation.

To carry out the experimental assays, a simulated gas stream was employed, which was produced by the stripping of pure liquid toluene; in short, a clean air stream, supplied continuously at a flow rate of 0.05 L/min, was bubbled into the washing bottle with 0.5 L of capacity (Duran from Normax Portugal) containing 0.3 L of pure liquid toluene. The stripping was performed at 5 °C (using a circulation bath CB 5–10 from Argo Lab) and atmospheric pressure, to yield a saturated toluene gas stream (see Figure 2). Then, this concentrated stream was mixed with another air stream (flow rate of 0.15 L/min), reaching a final toluene concentration of 0.10 g/L. The gas flow rate in each line was controlled using two direct-reading flowmeters (from Cole Palmer) coupled with non-return valves (from Swagelok); all gas flow rates were measured at room temperature and atmospheric pressure (25 °C and 1 atm). In its turn, the liquid phase present inside the reactor was composed of 0.3 L of distilled water (or tap water) with the pH adjusted to 3.0 (using 1 M H_2_SO_4_). Then, the desired and previously optimized amount of catalyst ([Fe^2+^] = 0.20 g/L) and oxidizing agent ([S_2_O_8_^2–^] = 1.70 g/L) were added to the reactor;^20^ simultaneously, the bubbling of the gas stream (gas flow rate of 0.20 L/min, measured at 25 °C and 1 atm) was initiated. This instant peak represents the start of the reaction (*t* = 0 min). All the runs were performed at room temperature and atmospheric pressure (25 °C and 1 atm).

Several samples were collected during the reaction time. In the case of the gas effluent, the samples were taken from the inlet and outlet streams of the reactor to quantify toluene; in the case of the liquid phase, the samples were taken to proceed with analyses of dissolved organic carbon (DOC), concentration of the persulfate remaining in the solution, and pH.

### 2.3 Analytical Methods

The quantification of toluene was performed using a gas chromatograph (GC) from Agilent Technologies (model 7820 A), equipped with a flame ionization detector (FID) and a capillary column (column HP-5MS from Agilent Technologies; 30 m of length, 0.250 mm of diameter, and 0.250 μm of film). Helium was used as carrier gas (25 mL/min, measured at 25 °C and 1 atm), and temperatures of 250, 150, and 250 °C were employed in the injector, oven, and detector, respectively. H_2_ and air were used in the FID detector at a flow rate of 40 and 290 mL/min, respectively. For toluene analysis, gas samples with 100 μL of volume were manually taken from the reactor inlet and outlet streams (see Figure 2) and injected in the GC using a gas-tight syringe (from Hamilton).

Dissolved organic carbon (DOC) in the water was determined through the catalytic oxidation of the liquid samples into CO_2_ at 720 °C; subsequently, the carbon dioxide was detected and quantified using an infrared detector. For that, a TOC-L analyzer equipped with a SHL autosampler (both from Shimadzu) was used—it was followed the analytic technique described in the method 5310D.^32^ IT should be noted that this method is not able to determine the volatile carbon fraction present in liquid samples.

Persulfate quantification was performed by using a spectrophotometric method according to the procedure described elsewhere.^33^ For that, the liquid sample was put in contact with several solutions previously prepared, namely, 10 mL of a 2.5 N H_2_SO_4_ solution, 0.1 mL of a 0.4 N FAS solution, and 0.2 mL of a 0.6 N NH_4_SCN solution. Then, after 40 min of contact, the intensity of the yellow-orange color of the complex formed was measured at 450 nm by using a Thermo Scientific spectrophotometer (model GENESYS 10S UV–Vis). The method’s interferences (caused by the presence of iron–catalyst—and by the solution pH) were taken into consideration; for that, the amount of iron added to the system as catalyst was discounted, and the pH effect was assessed by the use of some calibration curves covering the required ranges.^20^

The pH was measured according to the method 4500 H^+^ B,^32^ using a combination pH electrode (model SenTix 81) connected to a pH meter (model InoLab pH level 2), both from WTW.

### 2.4 Process Indicators

To evaluate the efficiency of the degradation process, two indicators were determined: the overall amount of toluene transferred per volume of liquid phase (η, mol/L; eq 5)^9^ and the enhancement factor (*E*; eq 6), the latter allowing to quantify the amount of toluene transferred as compared to a blank run, i.e., by simple absorption in the water.

$$\eta =\frac{Q_{\mathrm{gas}}C_{\mathrm{in}}\int_{0}^{t_{\mathrm{op}}}\left(1-\frac{C_{\mathrm{out}}(t)}{C_{\mathrm{in}}}\right)\mathrm{dt}}{{\mathrm{MM}}_{\mathrm{toluene}}V_{\mathrm{reactor}}}$$ 5

In eq 5, *C*_in_ and *C*_out_ represents the inlet and outlet toluene concentrations in the gas stream, respectively, in g/L; *Q*_gas_ is the gas fed flow rate, in L/min; MM_toluene_ represents the molecular weight of toluene, in g/mol; *V*_reactor_ is the volume of liquid phase inside the reactor (considering the sampling process), in L; and, *t*_op_ means the overall operation time, in min.

$$E=\frac{\eta_{\mathrm{experimental}\;\mathrm{test}}}{\eta_{\mathrm{absorption}}}$$ 6

As stated above, eq 6 gives information about the amount of toluene transferred in a given experiment (η_experimental test_) as compared to that by simple absorption (η_absorption_), into water, under the same operating conditions.

## 3 Results and Discussion

### 3.1 Effect of the Geometric Shape of the Diffuser

The study was initiated with a series of experimental assays performed in a BR, where it was intended to analyze the influence of the diffusers’ geometric shape in the process indicators. For that, two aquarium diffusers were tested: a spherical (Figure 3a) and a cylindrical (Figure 3b) one. The results obtained are gathered in Figure 4.

**Figure 3:**
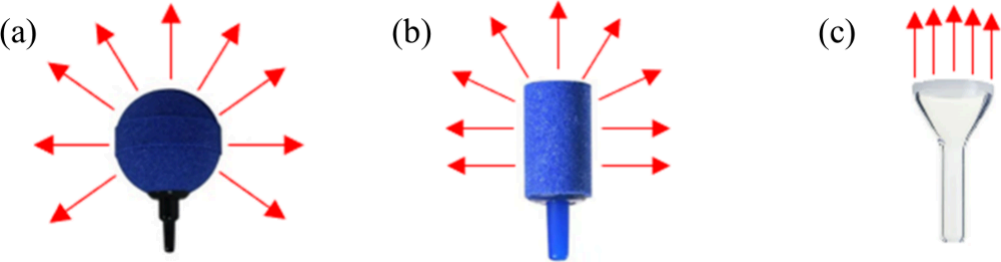
Spherical (a) and cylindrical (b) aquarium diffusers and diffuser plate (c). The red arrows describe the gas flow pattern established for each diffuser used in the present study.

**Figure 4:**
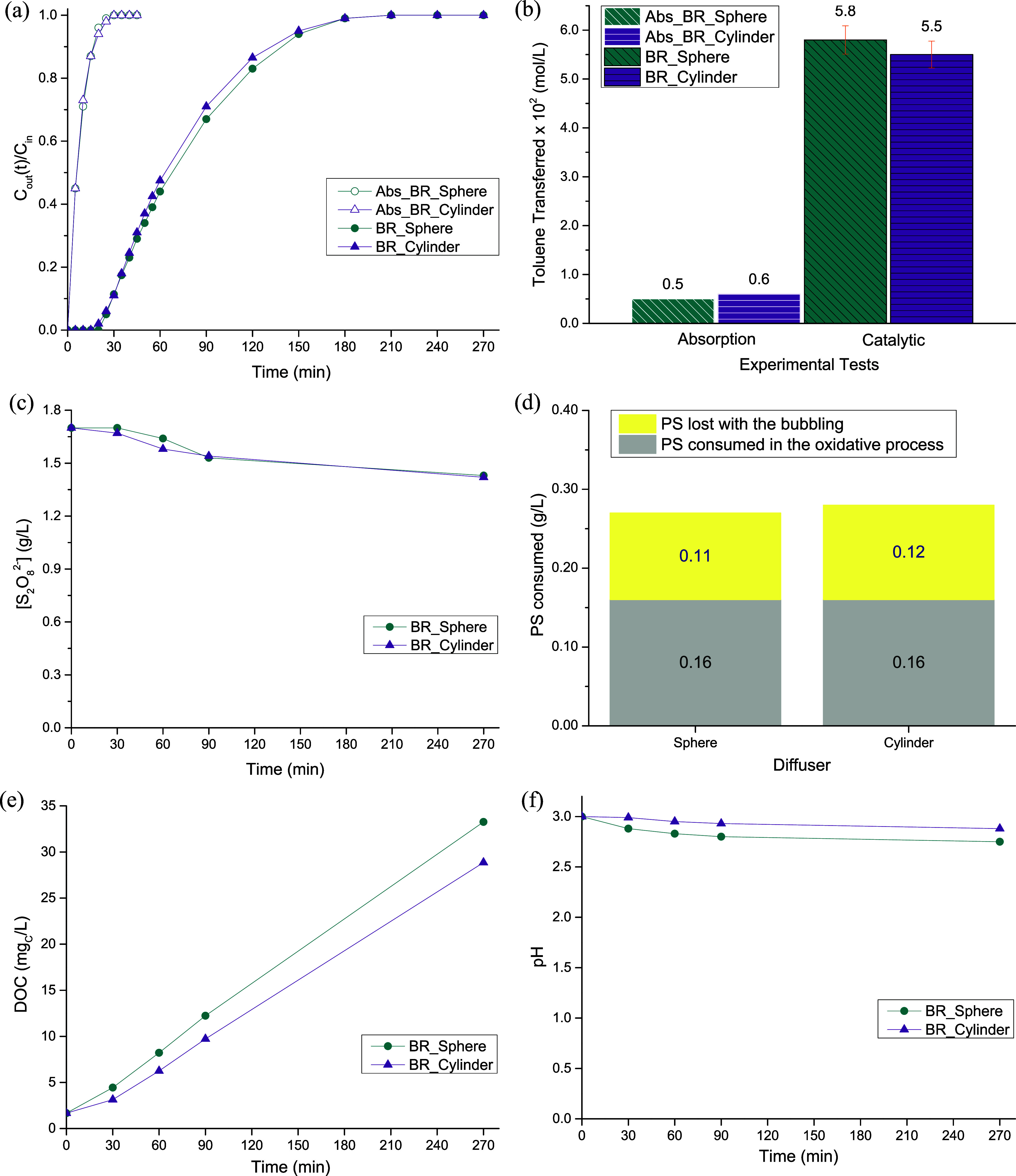
Toluene concentration in the treated gas stream along the reaction time (a), amount of toluene transferred from the gas stream to the liquid phase at the end of the process (b), persulfate concentration along the process (c), amount of persulfate consumed in the reaction (d), temporal evolution of the DOC (e), and pH of the liquid phase (f), for diffusers with different geometric shapes in the BR. Operating conditions: [S_2_O_8_^2–^]_initial_ = 1.70 g/L, [Fe^2+^] = 0.20 g/L, [Toluene]_in_ = 0.10 g/L, pH_initial_ = 3.0, *T* = 25 °C, V_reactor_ = 0.3 L, and Q_gas_ = 0.20 L/min (at 25 °C and 1 atm).

It should, however, be highlighted that in a subsequent section, a diffuser plate will also be tested (Figure 3c). As shown in Figure 3, depending on the type of diffuser used, whose geometry is different, the gas distribution within the liquid phase will be affected, namely, the gas flow pattern, which will consequently influence the pollutant mass transfer.

In Figure 4a, it is possible to see that all experimental curves present a very similar pattern: the concentration of toluene in the outlet stream increased along the reaction time until reaching the inlet concentration (i.e., *C*_out_/*C*_in_ = 1), the moment where the process was stopped because in such instant, the liquid is saturated with toluene. Moreover, it is possible to distinguish two types of experimental curves, the absorption and the reaction ones. The difference is that during the absorption runs (identified throughout this work as “Abs_”), none of the reagents were added to the liquid effluent, namely, PS and iron. The absorption tests were carried out to understand what happened if no treatment was conducted, and they were used as reference or control. Focusing on those assays, it was possible to see that they look very similar, with overlapping patterns for most of the time. This was corroborated by the amount of toluene that was transferred from the gas stream to the liquid effluent (see Figure 4b), which was determined by integrating the respective experimental curves (cf. eq 5), being both values were very alike and close to the reported solubility of toluene in water (of 0.540 g/L—that corresponds to 0.0059 mol/L—at 25 °C).^34^ In regard to the catalytic runs, it is possible to notice that by adding the reagents to the liquid phase, the performances achieved are very different from the ones obtained during the absorption experiments, the reaction tests being clearly extended over time, as a result of the presence of oxidizing species formed through the activation of PS (eqs 3 and 4). The process was prolonged from ca. 30 to ca. 210–240 min for both diffusers used (see Figure 4a), and inherently, the overall amount of toluene transferred increased from 0.005 to 0.058 mol/L when the spherical diffuser was used and from 0.006 to 0.055 mol/L with the cylindrical diffuser (see Figure 4b); this represents an improvement by a factor of 12 and 9, respectively (parameter *E*; calculated using eq 6). Because of that, and although both experimental curves seemed very similar, overlapping almost all the time, which resulted in very close values of toluene being transferred between the two effluents (difference of only 5%), the spherical aquarium diffuser was the one that was chosen to proceed with the experimental study. This similar performance showed that the geometric shape of the diffuser did not have a significant impact on the bubbles’ shape and size, which was corroborated experimentally by high-speed photography (see Section C of the Supporting Information—1. Influence of the geometric shape of the diffuser).

Regarding PS consumption, it is possible to state that the distinct geometric shapes of the aquarium diffusers also did not result in significant differences in the behavior found (see Figure 4c), being the total consumptions achieved pretty much the same (0.27 and 0.28 g/L, respectively, for the spherical and cylindrical diffusers—Figure 4d). Still, if only the amount of oxidizing agent consumed in the oxidative process was accounted, it is possible to notice that the same amount of PS was consumed—0.16 g/L with both diffusers; the amount of PS lost with the bubbling was determined in blank separate runs. However, persulfate was not completely consumed in any of the runs performed, remaining in solution 1.40 of the 1.70 g/L initially added to the system. One of the possible motives for the end of the process, despite the high amount of PS still available in the solution, is the fact that toluene could be transferred faster from the gas stream to liquid phase than the time that is needed for the sulfate radicals to be formed and degrade the model pollutant, i.e., the gas–liquid mass transfer occurs more quickly than the chemical reactions (that are rate-controlling), which leads to the saturation of the liquid effluent and, consequently, to the release of toluene by the outlet gas stream. Another explanation can be related to the organic compounds that are being accumulated in the liquid effluent along the reaction time as a consequence of toluene oxidation (Figure 4e), which will somehow inhibit and/or compete with toluene to be transferred and dissolved, as discussed by other authors.^9^

Figure 4e shows the amount of organic compounds that are dissolved within the liquid effluent, which are formed during toluene’s degradation. Slightly higher DOC values were obtained for the “BR_Sphere” as a result of the slightly higher transfer of toluene, which led to a higher accumulation of intermediate compounds in the liquid phase. However, if the final DOC values of both curves are compared (instant where both curves are more distant from each other), the difference is only about 4.4 mg_C_/L, which is not significant, allowing to consider similar intermediate compounds accumulation in both experiments.

Finally, Figure 4f presents the pH evolution observed during the experiments. It is expected a pH drop as the accumulation of organic compounds in the liquid effluent increases, because the main intermediates and more refractory compounds of toluene degradation are short-chain organic acids, i.e., carboxylic acids (namely, oxalic, maleic, oxamic, pyruvic, succinic, formic, acetic, and fumaric).^35,36^ Therefore, as the accumulation of organics was very similar in both experiments, similar pH evolution patterns were obtained (final pH values of 2.75 for the “BR_Sphere” test and 2.88 for the “BR_Cylinder” run).

### 3.2 Effect of Reactors’ Configuration

Using the spherical diffuser, in this section it was analyzed the impact of the configuration of the reactor used. The experimental conditions employed in the two tests were the same, being the only difference the type of reactor used—BR *vs.* BCR, with different aspect ratios (though the volume of liquid phase is the same). The results obtained are gathered in Figure 5.

**Figure 5:**
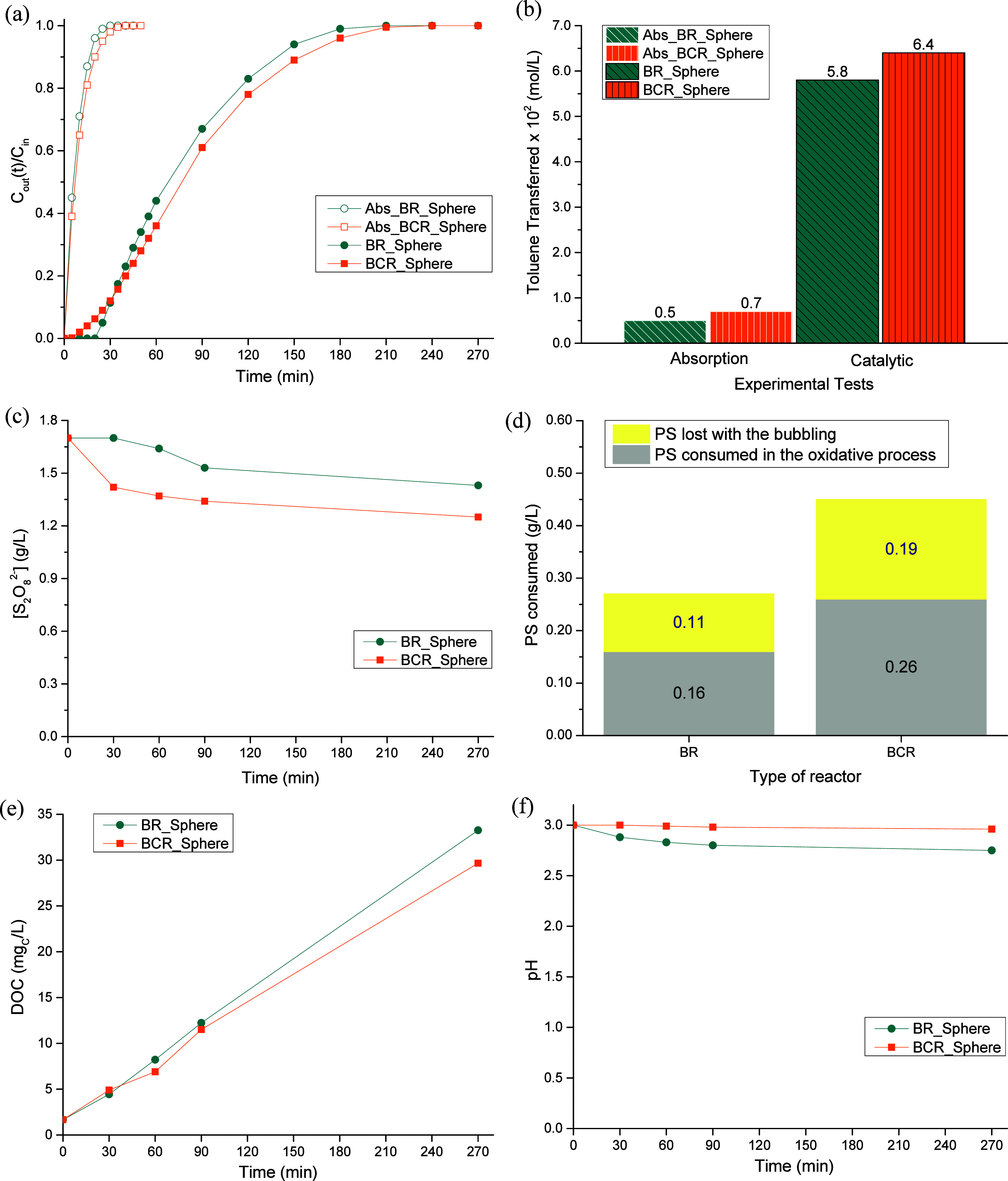
Toluene concentration in the treated gas stream along the reaction time (a), amount of toluene transferred from the gas stream to the liquid phase at the end of the process (b), persulfate concentration along the process (c), amount of persulfate consumed in the reaction (d), temporal evolution of the DOC (e), and pH of the liquid phase (f), for different reactor configurations (BR and BCR) with the spherical diffuser. Operating conditions: [S_2_O_8_^2–^]_initial_ = 1.70 g/L, [Fe^2+^] = 0.20 g/L, [Toluene]_in_ = 0.10 g/L, pH_initial_ = 3.0, *T* = 25 °C, V_reactor_ = 0.3 L, and Q_gas_ = 0.20 L/min (at 25 °C and 1 atm).

Focusing on the absorption curves (Figure 5a), it can be seen that the experiment performed in the BCR was slightly extended over time, meaning that toluene was detected in the outlet gas stream in a more advanced stage, which resulted in a slightly higher transfer of toluene (Figure 5b). As, at this point, none of the reagents were present in the liquid phase—water—this means that, only by changing the reactor’s configuration, the transfer of the pollutant was affected, which can be ascribed to the differences in reactors’ physical dimensions (Table 1) that affected the height of the liquid phase inside the reactor (*H*_liquid_) and, consequently, the aspect ratio *H*_liquid_/*D*_int_. In what concerns the catalytic runs, it is possible to see the occurrence of the same pattern previously analyzed: the experimental test performed in the BCR was extended over time, which resulted in a higher amount of toluene being transferred (0.064 mol/L, Figure 5b), representing an enhancement of 9 times regarding its absorption in water (parameter *E*; calculated using eq 6). According to other authors,^37^ improved decontamination factors have been observed with the increase of the liquid level height; when the level of liquid is increased inside the vessel, it takes more time for the bubbles to reach the surface of the liquid, increasing its residence time, and, consequently, the pollutant mass transfer between the gas and liquid phases.

From Figure 5c and 5d, it is possible to state that the highest consumption of persulfate was achieved for the run performed in the BCR, in line with the fact that this reactor configuration was the one where a higher transfer of toluene was observed. On the other hand, the percentage of PS consumed in the oxidative process was, for both reactor configurations, nearly the same, ca. 59%.

Figure 5e shows the accumulation of intermediate compounds formed during toluene’s degradation; both experimental tests presented similar behaviors (major difference of only 3.6 mg_C_/L, being obtained just before the interruption of the treatment process). However, since a slightly higher transfer of toluene was obtained on the “BCR_Sphere” run (and higher PS consumption in the oxidation process), it was likely to observe a higher accumulation of intermediate compounds in the solution for that experiment, something that did not happen; results obtained can be tentatively ascribed to a higher oxidation of the intermediate compounds in such run. Although the evolution of the intermediate compounds has not been followed along this study, several authors^4,6,35^ have experimentally shown in their works that a deeper oxidation can occur, being the intermediate compounds accumulated in the liquid phase degraded until the level of some volatile compounds or even carbon dioxide, which corroborate the behavior found in this work since they are not measured in DOC analysis.

The pH patterns (Figure 5f) are dependent on the reaction intermediates formed, the mechanism of which is rather complex. Additionally, the pH drop can also occur due to the formation of H^+^ species, because the degradative process takes place within the liquid phase (eqs 7 and 8)^38^ or because of the catalyst regeneration (eq 9).^16^ Therefore, and given the process complexity, one cannot infer about medium acidification based only on the DOC data, as other parallel reactions occur simultaneously.

$$S_{2}O_{8}^{2-}+H_{2}O\to 2{\mathrm{HSO}}_{4}^{-}+\frac{1}{2}O_{2}\to 2{\mathrm{SO}}_{4}^{2-}+2H^{+}+\frac{1}{2}O_{2}$$ 7

$${\mathrm{SO}}_{4}^{\cdot -}+H_{2}O\to {\mathrm{HO}}^{\cdot }+{\mathrm{HSO}}_{4}^{-}\to {\mathrm{HO}}^{\cdot }+{\mathrm{SO}}_{4}^{2-}+H^{+}$$ 8

$${\mathrm{Fe}}^{3+}+xH_{2}O\to \mathrm{Fe}{\left(\mathrm{OH}\right)}_{x}^{(3-x)+}+xH^{+}$$ 9

### 3.3 Effect of Type of Diffuser in the BCR

The best treatment performance was achieved when the BCR was used, and because of that, it was the reactor configuration selected to pursue the present study. With the main goal of understanding the influence of using different types of diffusers, the performance achieved by the spherical aquarium diffuser was compared with the one achieved by a diffuser plate (Figure 3c; from here on, it will be called *p3*, the commercial designation of the diffuser plate). The results obtained are gathered in Figure 6.

**Figure 6:**
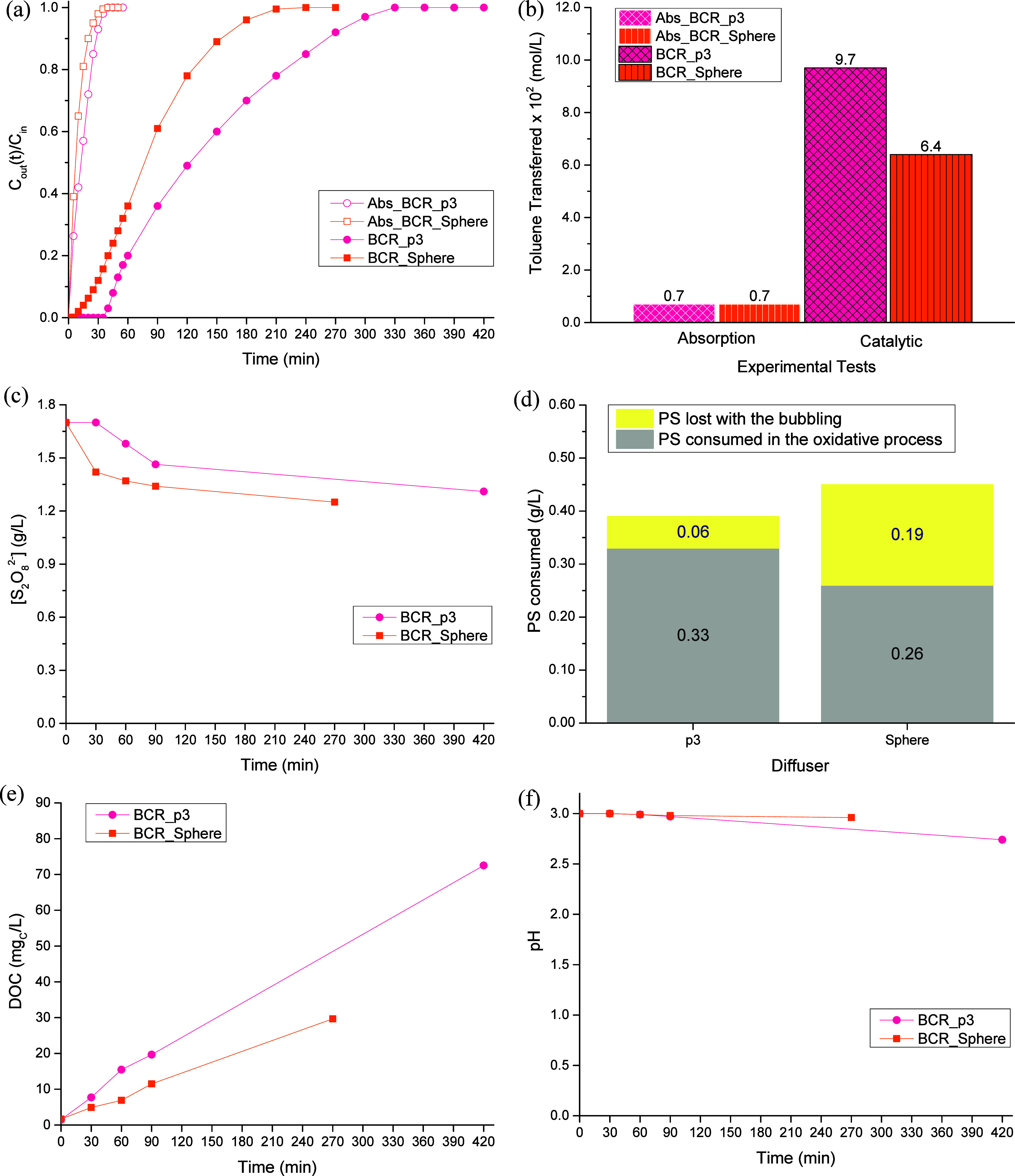
Toluene concentration in the treated gas stream along the reaction time (a), amount of toluene transferred from the gas stream to the liquid phase at the end of the process (b), persulfate concentration along the process (c), amount of persulfate consumed in the reaction (d), temporal evolution of the DOC (e), and pH of the liquid phase (f), for different types of diffusers in the BCR. Operating conditions: [S_2_O_8_^2–^]_initial_ = 1.70 g/L, [Fe^2+^] = 0.20 g/L, [Toluene]_in_ = 0.10 g/L, pH_initial_ = 3.0, *T* = 25 °C, V_reactor_ = 0.3 L, and Q_gas_ = 0.20 L/min (at 25 °C and 1 atm).

Considering the absorption curves (Figure 6a), almost no difference is seen for both diffusers, meaning that the amount of toluene transferred is very similar for both experiments (Figure 6b) and similar to the theoretical solubility of toluene in water. Regarding the catalytic runs, there is a notable difference between the two assays, with the “BCR_*p3*” test achieving the best treatment performance (amount of toluene transferred of 0.097 mol/L, which represents an enhancement of 14 times when compared to the solubility of toluene in water—eq 6). This can be ascribed to the smaller bubbles formed when the diffuser plate is used (Supporting Information Figure S4; the bubbles formed with the *p3* are smaller than the ones formed with the spherical diffuser, mainly in sections 2 and 3 of the BCR—central zones of the column). This maximizes the mass transfer process of the pollutant, due to the increase in the transfer area per volume unit (m^2^/m^3^); basically, smaller bubbles exhibit superior characteristics in terms of high gas–liquid mass transfer, being more efficient in increasing interfacial area and gas dissolubility.^39^

Regarding the consumption of PS, in Figure 6c, a higher total consumption of oxidizing agent is observed for the “BCR_Sphere” assay. However, if the amount of PS lost with the bubbling is considered (Figure 6d), it is possible to see that the amount of oxidizing agent that was, in fact, used in the decontamination of the gas stream was higher for the experimental run where the diffuser plate was used (i.e., the consumption of PS was more efficient when the plate diffuser was used), being the greatest loss of persulfate in the “BCR_Sphere” experiment related to the type of flow that the aquarium diffuser promotes.

The higher transfer of toluene from the gas stream to the liquid phase in the run where the diffuser plate was used, allied to the higher consumption of oxidizing agent for the chemical oxidation reactions, resulted in a larger accumulation of intermediate compounds inside the reactor (Figure 6e); even so, pH patterns are rather similar for the reasons stated in the previous section (Figure 6f).

### 3.4 Effect of Diffuser Porosity

As the use of the diffuser plate resulted in a much better treatment performance, this was the type of diffuser that was selected to pursue the study. There are available in the market diffusers with different levels of porosity, i.e., with holes with different dimensions, which will originate bubbles of different sizes; increased porosities mean smaller holes/smaller bubbles (see Section C of the Supporting Information—2. Effect of the type and porosity of the diffuser). To analyze the impact of the diffuser porosity on mass transfer and, consequently, on toluene’s degradation, three commercial diffuser plates were chosen (*p0*, *p2*, and *p3* according to Duran plate reference, whose designation was made by the industry in accordance with the porosity of the plate diffuser; see Table 2). The results obtained are collected in Figure 7.

**Table 2:** Commercial Plate Diffusers Were Selected to Be Used in the BCR

Diffuser plate	Diffuser holes’ diameter (μm)
*p0*	160–250
*p2*	40–100
*p3*	16–40

**Figure 7:**
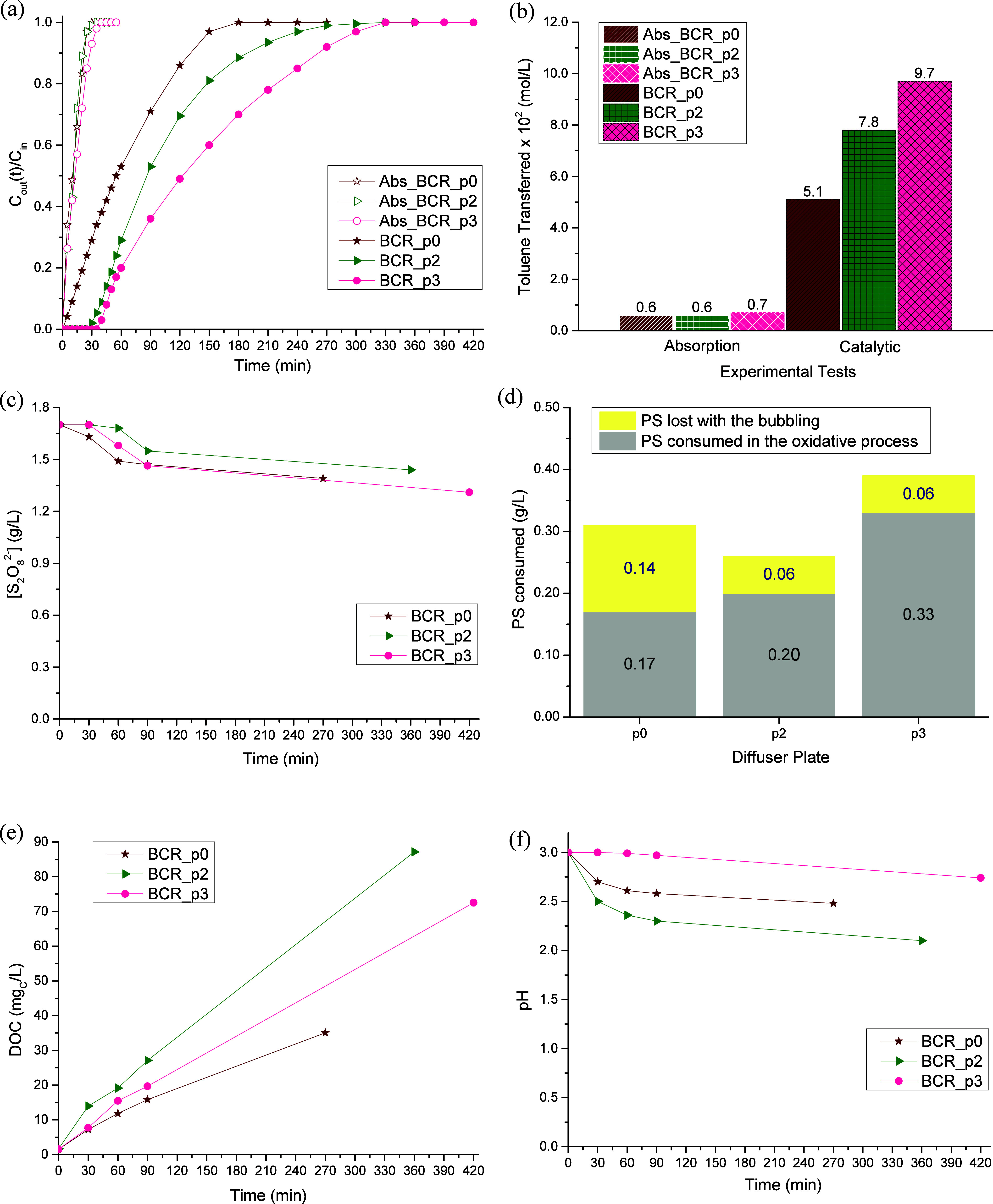
Toluene concentration in the treated gas stream along the reaction time (a), amount of toluene transferred from the gas stream to the liquid phase at the end of the process (b), persulfate concentration along the process (c), amount of persulfate consumed in the reaction (d), temporal evolution of the DOC (e), and pH of the liquid phase (f), for diffusers with different porosities in the BCR. Operating conditions: [S_2_O_8_^2–^]_initial_ = 1.70 g/L, [Fe^2+^] = 0.20 g/L, [Toluene]_in_ = 0.10 g/L, pH_initial_ = 3.0, *T* = 25 °C, V_reactor_ = 0.3 L, and Q_gas_ = 0.20 L/min (at 25 °C and 1 atm).

According to Figure 7a, the three absorption curves nearly overlap, resulting in similar amounts of toluene being transferred between the two phases (Figure 7b). Regarding the catalytic runs, it is possible to notice a displacement of the curves in time as the porosity of the diffuser increases, being the best treatment performance obtained with the “BCR_*p3*” assay. Because the bubble size decreases as the plate porosity increases (see Supporting Information Figures S4 and S5), the improvement of the process performance can be ascribed to the maximization of the mass transfer process.

Regarding the consumption of PS (Figure 7c), it is possible to see that there is not a clear pattern, being similar in all runs. A relationship started to appear when the amount of PS that goes out of the system with the air bubbling is considered (Figure 7d). During this analysis, it was possible to verify that as the porosity of the diffuser plate increases, the amount (in %) of PS that leaves the treatment system decreases (*p0*: 45%, *p2*: 22%, and *p3*: 15%); so, discounting this amount from the total amount of PS consumed in each experiment, it is possible to know the real amount of oxidizing agent that was consumed in the oxidative catalytic process. Data obtained show that from *p0* to *p3*, more PS is effectively used and consumed in the desired oxidation process.

Regarding the accumulation of intermediate compounds (Figure 7e), an increase in the DOC was clearly observed when comparing *p0* to *p2*, in line with the PS consumed in the oxidative process. Such increase in DOC leads to a higher medium acidification (Figure 7f), ascribed to the formation of carboxylic acids. Such trend is however not observed for *p3*. With this diffuser plate, a much higher PS consumption was noticed, which can be responsible for a deeper oxidation of the reaction intermediates (and particularly of the end-products—carboxylic acids) and inherently a not so pronounced increase in DOC and pH drop.

### 3.5 Effect of Water Matrix

The water matrix present inside the BCR may have some impact on the mass transfer process of the pollutant and, consequently, on the treatment process. So, to understand how the liquid phase used affects the decontamination of the gas stream, the distilled water used until now was replaced by tap water (the ion composition of both waters was analyzed, and the information is shown in Table 3). The results obtained are presented in Figure 8.

**Table 3:** Water Matrixes Composition

Ion	Distilled water (ppm)	Tap water (ppm)
Fluoride	0.02	0.04
Chloride	0.34	14.12
Nitrate	0.10	2.29
Bromide	<0.10	0.27
Phosphate	<0.10	32.08
Potassium	0.04	0.03
Calcium	0.02	16.17
Magnesium	0.03	2.17

**Figure 8:**
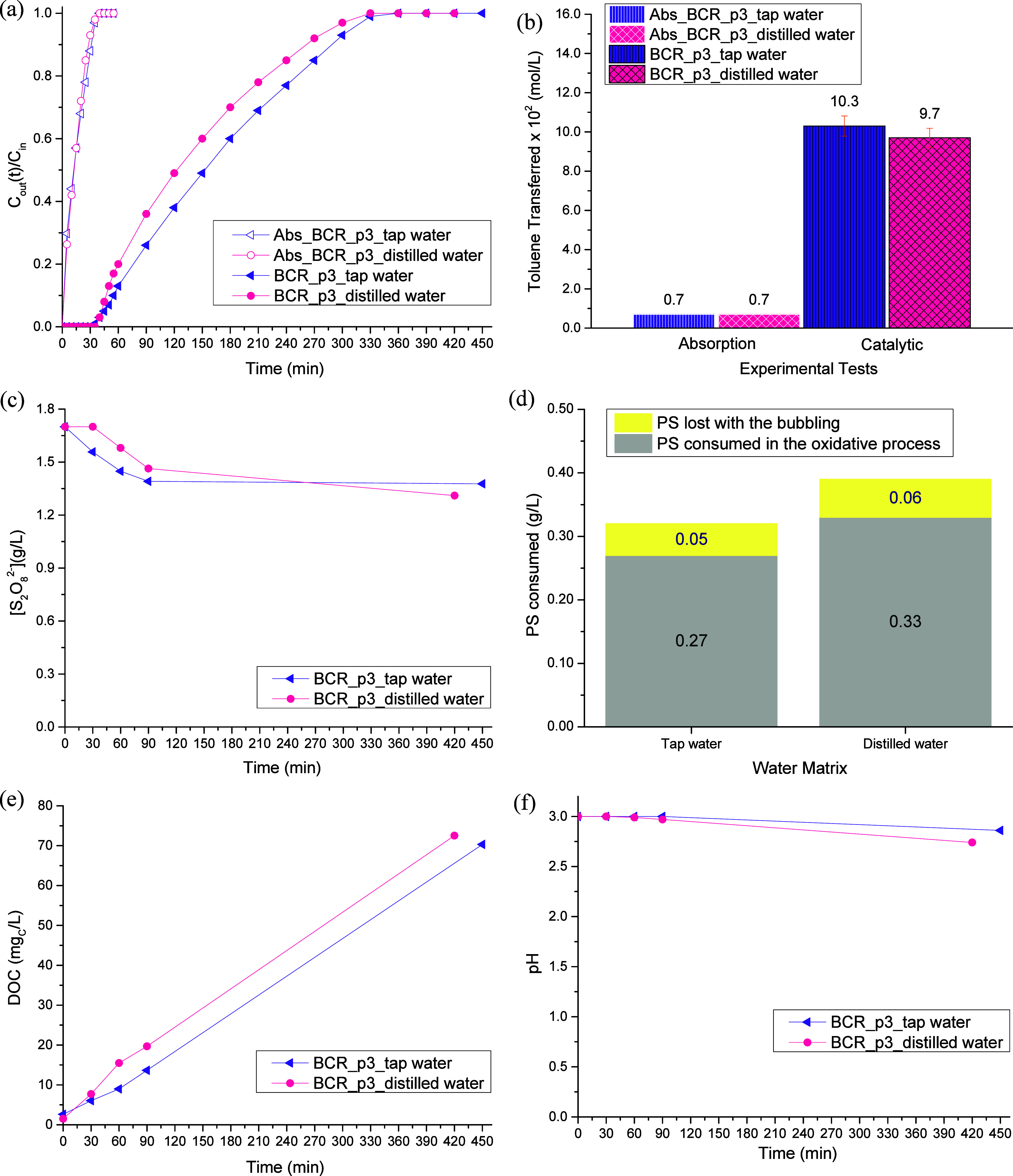
Toluene concentration in the treated gas stream along the reaction time (a), amount of toluene transferred from the gas stream to the liquid phase at the end of the process (b), persulfate concentration along the process (c), amount of persulfate consumed in the reaction (d), temporal evolution of the DOC (e), and pH of the liquid phase (f), for different water matrixes in the BCR with the *p3* diffuser. Operating conditions: [S_2_O_8_^2–^]_initial_ = 1.70 g/L, [Fe^2+^] = 0.20 g/L, [Toluene]_in_ = 0.10 g/L, pH_initial_ = 3.0, *T* = 25 °C, V_reactor_ = 0.3 L, and Q_gas_ = 0.20 L/min (at 25 °C and 1 atm).

In Figure 8a, it is possible to see that both absorption curves overlap, resulting in similar amounts of toluene transferred between the gas stream and the liquid phase (Figure 8b), and again close to the value of the solubility of toluene in water. Regarding the catalytic curves, it is possible to notice that the “BCR_*p3*_tap water” assay presents a slight displacement in time, which resulted in the transfer of 0.103 mol/L of toluene (Figure 8b), representing an improvement by a factor of 15 (*E*; using eq 6) when compared to the respective absorption in water. However, comparing both experimental tests, the difference obtained was only about 5% (which can be ascribed to experimental uncertainty), indicating that the type of water used did not have a significant impact on toluene’s transfer and degradation, despite their different composition (Table 3). The presence of some ions in tap water (as phosphate, for example—the highest ion concentration found in the tap water sample analyzed; see Table 3) could impact the AOP performance, due to their influence on sulfate radicals’ formation/scavenging; however, such impact was not observed in the present study. The same behavior was found by other authors,^40^ who stated that the presence of phosphate did not affect toluene’s oxidation due to its weak strength as a scavenger, resulting in a slow rate of the scavenging reaction of sulfate radicals. Therefore, for future applications, tap water can be used as the liquid phase without any problems, which is a benefit regarding the industrial application of the present treatment process.

In what concerns the liquid samples, it is possible to notice that (i) the consumption of persulfate (Figure 8c and 8d); (ii) the accumulation of intermediate compounds within the liquid effluent (Figure 8e); and (iii) the evolution of pH (Figure 8f) were very similar for both experimental tests, corroborating the fact that the water matrix has no impact on toluene’s mass transfer and on its further oxidation by this AOP.

## 4 Future Work

As future work, the authors propose the following:

- Analyze in greater detail both the gas outlet stream and the liquid effluent. This further investigation will also allow to get further insights about the reaction mechanism, and reaction by-products present in each phase, if any;
- Perform gas–liquid treatment cycles in order to get the technology under study closer to the industrial reality, namely, in what concerns the continuous operation, the dosage of oxidizing agent (decrease the dose of PS used), and the concentration of pollutant (decrease the concentration of toluene—in most industries, toluene concentration in gas streams ranges between 500 and 1500 mg/m^3^ ^41^);
- Test the BCR application to real gas streams, with other pollutants and impurities.

## 5 Conclusions

A toluene-containing gas stream was decontaminated by application of an activated persulfate-based advanced oxidation process, being the main goal to better understand the impact that some parameters of the reactor and diffuser have on the mass transfer process of the pollutant from the gas to the liquid and, consequently, on its degradation.

During the present work, it was analyzed the influence of (i) the reactors’ configuration (aspect ratio); (ii) the diffusers used (geometric shape—spherical *vs*. cylindrical; type—aquarium *vs*. diffuser plate; and their porosity—with orifice size ranging from 16 to 250 μm); and (iii) the water matrix (distilled *vs*. tap water) on toluene’s transfer, degradation, and organics mineralization. The treatment performance was maximized when the bubble column reactor (with a higher *H*_liquid_/*D*_int_ aspect ratio) and the diffuser plate with the highest porosity (smaller bubbles’ size) were used. Regarding the water matrix, the use of distilled or tap water seems not to have a significant impact on toluene’s degradation, meaning that the latter can be successfully used, which is an advantage for the industrial application of the treatment technology under study.

## Supplementary Material



## Notes

Drawbacks of some treatment methods applied to decontaminate toluene-containing gas streams; studies focused on activated persulfate-based oxidation for the degradation of toluene-containing gas streams; determination of the bubbles’ size: (i) influence of the geometric shape of the diffuser and (ii) effect of the type and porosity of the diffuser (PDF)
