# Supplementary material for: Toluene-Containing Gas Stream Treatment by Persulfate-Based Oxidation: Process Variables Affecting Mass Transfer
Source: Ind Eng Chem Res. 2025 Aug 29;64(36):17281–93. doi: 10.1021/acs.iecr.5c00924 (PMC12821061; doi:10.1021/acs.iecr.5c00924)
Supplement: Supplementary file 1 [file ie5c00924_si_001.pdf]

Supporting Information Section

Toluene-containing gas stream treatment by persulfate-based oxidation: process variables affecting mass transfer

Ana S. P. Alves[1,2], Carmen S. D. Rodrigues[1,2], João M. Miranda[2,3] and Luís M. Madeira[1,2,*]

[1] LEPABE – Laboratory for Process Engineering, Environment, Biotechnology and Energy, Faculty of Engineering, University of Porto, Rua Dr. Roberto Frias, 4200-465 Porto, Portugal.

[2] ALiCE – Associate Laboratory in Chemical Engineering, Faculty of Engineering, University of Porto, Rua Dr. Roberto Frias, 4200-465 Porto, Portugal.

[3] CEFT – Transport Phenomena Research Center, Faculty of Engineering, University of Porto, Rua Dr. Roberto Frias, 4200-465 Porto, Portugal.

[*] Corresponding author - Tel.: +351 22 041 3623; E-mail: mmadeira@fe.up.pt

Section A - Drawbacks of some treatment methods applied to decontaminate toluene-containing gas streams

Treatment process	Cons	Reference
Catalytic ozonation	↓ Oxidation process carried out by ozone molecules, which can be corrosive;<br>↓ During the treatment process can be produced by-products potentially carcinogenic;<br>↓ It is a very expensive process (ozone generation requires high energy and must be generated on-site; and ozone units require a high cost of operation and maintenance).	[1]
Adsorption	↓ There is no real destruction of the organic compound(s), being only transferred from one phase to another.	[2]
Fenton and photo-Fenton reactions	↓ Degradative process carried out by hydroxyl radicals ($OH^{\cdot}$), which according to some authors are less efficient in dealing with effluent's decontamination when compared to sulfate radicals;<br>↓ Although hydroxyl and sulfate radicals have a similar redox potential (2.80 eV for $HO^{\cdot}$ and 2.60 eV for $SO_4^{\cdot-}$), the first ones are less stable and have a lower half-life time ($2x10^{-8}$ s for $HO^{\cdot}$ *vs.* $3\text{-}4x10^{-5}$ s for $SO_4^{\cdot-}$), which contributes to a less efficient treatment of the target pollutant(s);<br>↓ Hydroxyl radicals are less reactive when dealing with organic compounds that have unsaturated bonds or aromatic constituents (as is the case of toluene);<br>↓ Hydroxyl radicals are easily captured by other species that may be present within the effluent, such as natural organic matter and bicarbonates, for example, increasing the extent of parallel and undesired scavenging reactions;<br>↓ Processes not applicable in a wide pH range; more efficient at low pH.	[3-8]

Treatment process	Cons	Reference
Biofiltration	↓ Not feasible for non-biodegradable or toxic substances, once they may be inhibited by the properties of some VOCs;<br>↓ VOCs removal can be easily affected by residence time, temperature, nutrients concentration, pH and salinity; apart from by VOCs' structure and properties; or even, by microbial community structure;<br>↓ There are some problems associated with the excessive accumulation of biomass during the process, which can lead to the blockage of the packed bed and increase the pressure drop and energy consumption.	[2]
Dielectric barrier discharge	↓ Requires electric current to generate energy in the battery;<br>↓ Produce ozone.	[9]

Section B - Studies focused on activated persulfate-based oxidation for the degradation of toluene-containing gas streams

Gaseous Pollutant(s)	Operating Conditions	Removals	Reference
**Toluene**	$[VOC]_{inlet}$ = 30 ppm; [PMS] = 2 g/L; Catalyst ($CoFe_2O_4/MoS_2$) dosage = 0.1 g/L; T = 30 ºC; pH = 7.0; t = 2 h	Removal efficiency of 95%	[10]
	$[VOC]_{inlet}$ = 30 ppmv; [PMS] = 3 g/L; Catalyst (Co-Fe) dosage = 0.2 g/L; T = 25 ºC; pH = 7.0	Removal efficiency above 95%	[11]
	$[VOC]_{inlet}$ = 30 ppmv; [PDS] = 3 mM; Catalyst ($KMnO_4$) dosage = 0.3 mM; pH = 4.6; UV = 254 nm (4 W UV light lamp); t = 4 h	Removal efficiency above 90%	[12]
	$[VOC]_{inlet}$ = 1 mM; [PDS] = 18 mM; T = 40 ºC; pH = 7.0; t = 564 h	Complete mineralization (~97%)	[13]
**Toluene** and **Ethyl acetate**	$[VOC]_{inlet}$ = 30 ppmv; [PMS] = 9 mM; pH = 3.0; UV = 254 nm (4 W UV light lamp); t = 150 min	Maximum removal efficiencies of 96.5 and 98.3%, respectively	[14]
**Styrene** (also **Toluene**, **Benzene** and **Ethyl acetate**)	$[VOCs]_{inlet}$ = 30 ± 2 ppmv; [PMS] = 5 mM; [MWCNTs] = 0.08 g/L; t = 2 h	Removal efficiencies of ~98, ~90, ~90 and 100%, respectively<br>Average mineralization rates of 76, 88, 75 and 66%, respectively	[15]

Gaseous Pollutant(s)	Operating Conditions	Removals	Reference
**Chlorobenzene**, **Styrene** and **Toluene**	$[VOC]_{inlet}$ = 15 ppmv; [PMS] = 1.6 mM; $[Fe^{3+}]$ = 0.50 mM; m MoS_2 = 15 mg; T = 25 ºC; pH = 7.0 ; t = 120 min	Removal efficiencies of 90, 97 and 90%, respectively<br>Average removal efficiency of the mixture = 90%	[16]
**Benzene** (also **Toluene**, **Ethylbenzene** and **Xylene**)	$[VOC]_{inlet}$ = 300 ppm; [PMS] = 3.5 mg/min; Catalyst ($MnCoO_x$/Kaolin) dosage = 1.4 g/L; T = 25 ºC; Q_{gas} = 100 mL/min; t = 120 min	Removal rates of 98, 99, 91 and 90%, respectively<br>Average removal rate of the mixture of 99%	[17]
**BTEX** (**B**enzene, **T**oluene, **E**thylbenzene and *o*-**X**ylene)	$[VOC]_{inlet}$ = 40 mg/L; r_{ox} = 5; [Asph] = 0.5 g/L; T = 25 ± 2 ºC; pH = 5.0; t = 360 min; US = 20 – 40 kHz	PDS/US/Asph: removals of 78, 94, 98 and 98%, respectively<br>PMS/US/Asph: removals of 76, 91, 97 and 97%, respectively	[18]
	$[Benzene]_{inlet}$ = 400 mg/L; $[Toluene]_{inlet}$ = 143 mg/L; $[Ethylbenzene]_{inlet}$ = $[Xylene]_{inlet}$ = 570 mg/L; [PDS] = 0.1 M; t = 60 min; $[Fe^{2+}]$ = 1 M; Fe^{2+} pumping rate = 1.4 mL/min; Fe^{2+}/citric acid molar ratio = 5/3	~50% removal of BTEX gases	[19]

PMS - peroxymonosulfate; PDS - peroxydisulfate; MWCNTs - multiwalled carbon nanotubes; Asph - asphaltenes; r_{ox} - molar ratio of oxidant to BTEX, i.e., [PMS or PMS]:[BTEX].

Section C - Determination of the bubbles' size

When a bubble reactor (BR) or a bubble column reactor (BCR) is used, the size of the bubbles formed plays a very important role in the treatment process (namely in the one used in this work) as it defines the interfacial area available for mass transfer and, consequently, the pollutant removal efficiency [20]. Because of that, the bubbles' size and their distribution along the reactor were determined.

In the past, many different methods were used for the measurement of bubbles' size. Electrical resistance tomography, local optical probes, particle image velocimetry, acoustic bubble spectrometer and high-speed photographic techniques, are some of them [21], being the nonintrusive technologies the most frequently employed since they do not interfere in fluid dynamics [21]. High-speed photography was the method selected to pursue with the present study given its ease of use.

The photographic technique was adopted with the main goal of experimentally determining the mean diameter of the bubbles, for each diffuser used (*cf*, Figure 3). For that, a high-speed camera was used. The images obtained were then processed by using ImageJ software. Auto processing of the images is not possible in ImageJ, so the bubble diameter was estimated manually drawing a line on the bubble, which, after comparison with a known distance (scale), allowed the determination of the experimental diameter of each bubble (d_{exp}). In the case of the BR, the dimensions of the diffuser were used as a scale, whereas in the BCR it was used the internal diameter of the reactor (see Figure S1 a) and b), respectively). To reduce the measurement error, 100 bubbles (*n*) were analyzed by the image processing software and the mean diameter of the bubbles ($d_{average}$, *cf*. Equation S1) was determined.

a) b)

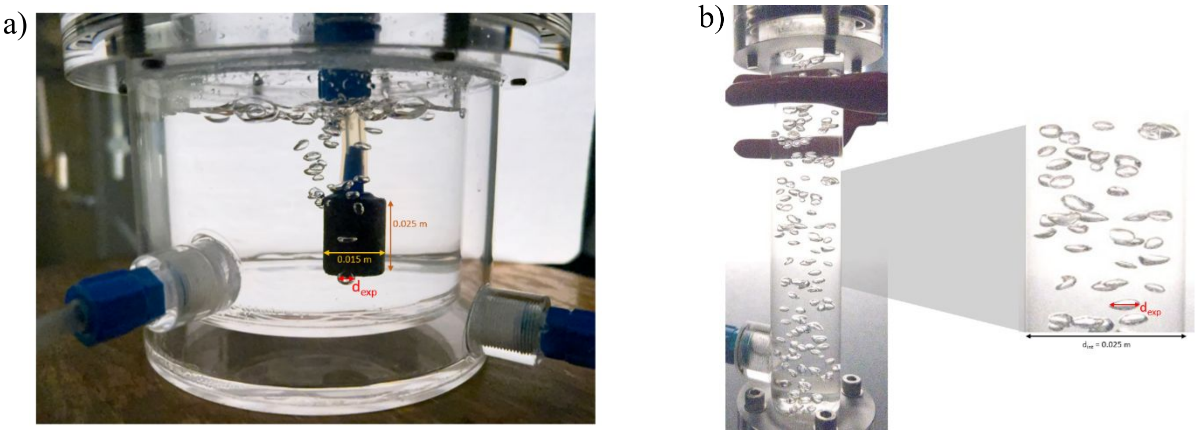

Figure S1. Bubble measurement in the a) bubble reactor (BR) and b) bubble column reactor (BCR).

$$d_{average} = \frac{\sum_{i=1}^{n} d_{exp}}{n} \quad \text{(S1)}$$

1. Influence of the geometric shape of the diffuser

In the BR, it was intended to analyze the influence of the geometric shape of the diffuser on the bubbles' size and form. The results obtained are gathered in Figures S2.

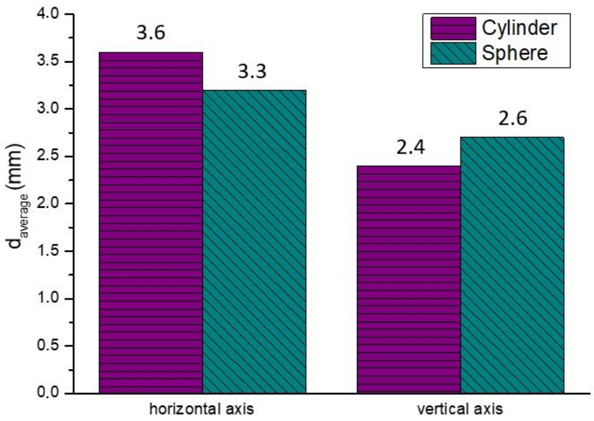

Figure S2. Dimension of the bubbles formed in the BR, using diffusers with different geometric shapes.

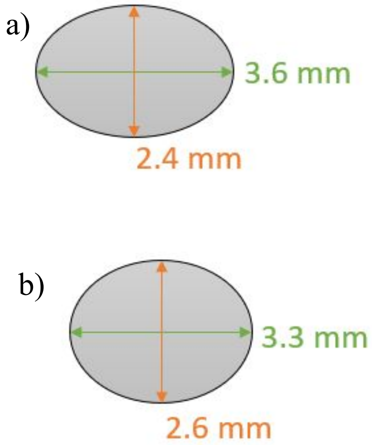

Figure S3. Bubbles formed by the a) cylindrical and b) spherical diffusers.

From Figure S2 it is possible to state that the bubbles seem to be a little bit influenced by the geometric shape of the aquarium diffuser used. The bubbles formed from the cylinder diffuser seem more flattened than the bubbles formed from the spherical diffuser (see Figure S3). However, the difference is very slight (less than 10%) and can be ascribed to the experimental error associated with the method selected. Consequently, it is possible to conclude that the geometric shape of the diffuser does not have a significant impact on bubbles' size and form, i.e., the bubbles are pretty similar.

2. Effect of the type and porosity of the diffuser

In the BCR, it was intended to analyze the influence of the type of the diffuser used and its porosity on bubbles' size, apart from their distribution along the reactor's height. For that the BCR was divided into four (equal) parts, numbered from the bottom to the top. The results obtained are summarized in Figure S4.

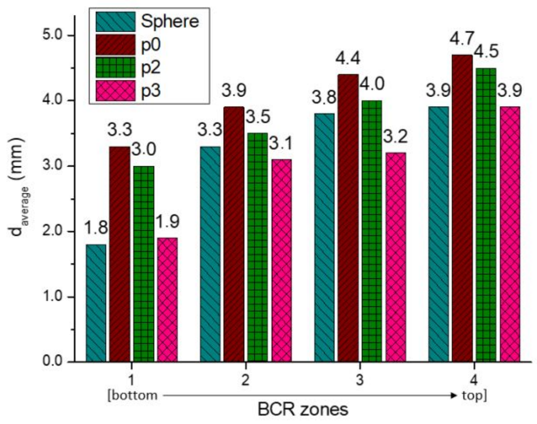

Figure S4. Dimension of the bubbles along the BCR, using different diffusers.

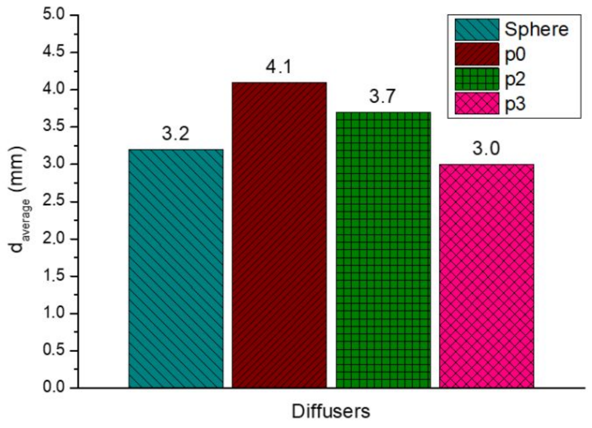

Figure S5. Average size of the bubbles when a BCR and different diffusers are used.

From Figure S4, it is possible to see that, for each diffuser used, the size of the bubbles increases along the reactor, which can be ascribed to the coalescence phenomena that occur over the bubble column length. Moreover, the bubbles formed when a *p0* diffuser plate (lower porosity, or higher diffuser holes' diameter) is used are bigger than the ones formed when a *p2* or a *p3* diffuser plate is used, which is in accordance with the porosity information present in Table 2. This can be more easily seen in Figure S5, where the $d_{average}$ obtained (if the four zones of the BCR were considered) are shown, i.e., along all the BCR's height. Lastly, it is still possible to notice that the size of the bubbles formed when the spherical aquarium diffuser is used is pretty similar to the size of the bubbles formed when the *p3* diffuser plate is used (error inferior to 10%) if the zones 1 and 4 are considered; however, in zones 2 and 3 the bubbles produced by the diffuser plate (*p3*) are smaller. This will certainly influence the mass transfer process of the pollutant from the gas stream to the liquid phase, once it influences the interfacial area available (m^2/m^3).

References

[1] Hameed, E. A.; Al-Falahi, H. A. F. The Quality of Drinking Wat between Ozone Sterilization Technology and Home Treatment System in Kirkuk Governorate. *IOP Conf. Ser. Mater. Sci. Eng.* 2021, 1094, 1-10. DOI: https://doi.org/10.1088/1757-899X/1094/1/012003.

[2] Lima, V. N.; Rodrigues, C. S. D.; Borges, R. A. C.; Madeira, L. M. Gaseous and liquid effluents treatment in bubble column reactors by advanced oxidation processes: A review. *Crit. Rev. Environ. Sci. Technol.* 2018, 48, 949-996. DOI: https://doi.org/10.1080/10643389.2018.1493335.

[3] He, L.; Chen, H.; Wu, L.; Zhang, Z.; Ma, Y.; Zhu, J.; Liu, J.; Yan, X.; Li, H.; Yang, L. Synergistic heat/UV activated persulfate for the treatment of nanofiltration concentrated leachate. *Ecotoxicol. Environ. Saf.* 2021, 208, 111522-111529. DOI: https://doi.org/10.1016/j.ecoenv.2020.111522.

[4] Li, J.; Ji, Q.; Lai, B.; Yuan, D. Degradation of *p*-nitrophenol by Fe^0/H_2O_2/persulfate system: Optimization, performance and mechanisms. *J. Taiwan Inst. Chem. Eng.* 2017, 80, 686-694. DOI: https://doi.org/10.1016/j.jtice.2017.09.002.

[5] Feng, Q.; Zhou, J.; Luo, W.; Ding, L.; Cai, W. Photo-Fenton removal of tetracycline hydrochloride using $LaFeO_3$ as a persulfate activator under visible light. *Ecotoxicol. Environ. Saf.* 2020, 198, 110661-110668. DOI: https://doi.org/10.1016/j.ecoenv.2020.110661.

[6] Yang, L.; Bai, X.; Shi, J.; Du, X.; Xu, L.; Jin, P. Quasi-full-visible-light absorption by D35-TiO_2/g-C_3N_4 for synergistic persulfate activation towards efficient photodegradation of micropollutants. *Appl. Catal. B: Environ.* 2019, 256, 117759-117774. DOI: https://doi.org/10.1016/j.apcatb.2019.117759.

[7] Sabri, M.; Habibi-Yangjeh, A.; Chand, H.; Krishnan, V. Activation of persulfate by novel TiO_2/FeOCl photocatalyst under visible light: Facile synthesis and high photocatalytic performance. *Sep. Purif. Technol.* 2020, 250, 117268-117281. DOI: https://doi.org/10.1016/j.seppur.2020.117268.

[8] Ahmadian, M.; Pirsaheb, M.; Janjani, H.; Hossaini, H. Ultraviolet activated persulfate based AOP for MTBE decomposition in aqueous solution. *Desalin. Water Treat.* 2019, 161, 269-274. DOI: https://doi.org/10.5004/dwt.2019.24310.

[9] Qin, C.; Guo, M.; Jiang, C.; Yu, R.; Huang, J.; Yan, D.; Li, S.; Dang, X. Simultaneous oxidation of toluene and ethyl acetate by dielectric barrier discharge combined with Fe, Mn and Mo catalysts. *Sci. Total Environ.* 2021, 782, 146931-146942. DOI: https://doi.org/10.1016/j.scitotenv.2021.146931.

[10] Zhang, X.; Zhang, W.; Zhang, X.; Li, J.; Wang, T.; Fan, Q.; Zhu, H.; Yang, Z.; Kong, C. Deep mineralization of VOCs in an embedded hybrid structure $CoFe_2O_4/MoS_2$/PMS wet scrubber system. *iScience*. 2023, 26, 108054-108068. DOI: https://doi.org/10.1016/j.isci.2023.108054.

[11] Xie, X.; Xie, R.; Suo, Z.; Huang, H.; Xing, M.; Lei, D. A highly dispersed Co-Fe bimetallic catalyst to active peroxymonosulfate for VOC degradation in a wet scrubber. *Environ. Sci. Nano*. 2021, 8, 2976-2987. DOI: https://doi.org/10.1039/d1en00547b.

[12] Xie, R.; Suo, Z.; Guo, K.; Feng, F.; Lan, B.; Zhang, T.; Li, G.; Huang, H. Promoting multiple reactive oxygen species generation for deep oxidation of VOCs by UV/persulfate/permanganate. *Sep. Purif. Technol.* 2023, 325, 124770-124779. DOI: https://doi.org/10.1016/j.seppur.2023.124770.

[13] Kalogerakis, G. C.; Boparai, H. K. ; Sleep, B. E. The journey of toluene to complete mineralization via heat-activated peroxydisulfate in water: intermediates analyses, CO_2 monitoring, and carbon mass balance. *J. Hazard. Mater.* 2022, 440, 129739-129754. DOI: https://doi.org/10.1016/j.jhazmat.2022.129739.

[14] Xie, R.; Ji, J.; Guo, K.; Lei, D.; Fan, Q.; Leung, D. Y. C.; Huang, H. Wet scrubber coupled with UV/PMS process for efficient removal of gaseous VOCs: Roles of sulfate and hydroxyl radicals. *Chem. Eng. J.* 2019, 356, 632-640. DOI: https://doi.org/10.1016/j.cej.2018.09.025.

[15] Wu, J.; Wang, J.; Liu, C.; Nie, C.; Wang, T.; Xie, X.; Cao, J.; Zhou, J.; Huang, H.; Li, D.; Wang, S.; Ao, Z. Removal of Gaseous Volatile Organic Compounds by a Multiwalled Carbon Nanotubes/Peroxymonosulfate Wet Scrubber. *Environ. Sci. Technol.* 2022, 56, 13996-14007. DOI: https://doi.org/10.1021/acs.est.2c03590.

[16] Xie, X.; Xiang, Y.; Cao, J.; Dai, W.; Ao, Z.; Huang, H.; Yang, X.; Xiao, F.; Ye, X. High-efficiency destruction of aromatic VOC mixtures in a MoS_2 cocatalytic Fe^{3+}/PMS reaction. *Sep. Purif. Technol.* 2023, 305, 122444-122454. DOI: https://doi.org/10.1016/j.seppur.2022.122444.

[17] Cheng, Z.; Zhou, Y.; Zhao, X.; Chen, Z.; Zhang, S.; Zhu, Z.; Zhou, Y.; Yang, Y.; Qi, J.; Li, J. Efficient removal of VOCs emission from soil thermal desorption via $MnCoO_x$/Kaolin activating peroxymonosulfate in wet scrubber. *Chem. Eng. J.* 2024, 480, 148159-148167. DOI: https://doi.org/10.1016/j.cej.2023.148159.

[18] Fedorov, K.; Plata-Gryl, M.; Khan, J. A.; Boczkaj, G. Ultrasound-assisted heterogeneous activation of persulfate and peroxymonosulfate by asphaltenes for the degradation of BTEX in water. *J. Hazard. Mater.* 2020, 397, 122804-122811. DOI: https://doi.org/10.1016/j.jhazmat.2020.122804.

[19] Liang, C.; Chen, Y.-J.; Chang, K.-J. Evaluation of persulfate oxidative wet scrubber for removing BTEX gases. *J. Hazard. Mater.* 2009, 164, 571-579. DOI: https://doi.org/10.1016/j.jhazmat.2008.08.056.

[20] Ahad, J.; Farooq, A.; Ahmad, M.; Waheed, K.; Qureshi, K. R.; Siddique, W.; Irfan, N. Performance of bubble column for iodine removal in a lab scale setup of filtered containment venting system. *Prog. Nucl. Energy.* 2023, 159, 104670-104679. DOI: https://doi.org/10.1016/j.pnucene.2023.104670.

[21] Zeng, W.; Jia, C.; Luo, H.; Yang, G.; Yang, G.; Zhang, Z. Microbubble-Dominated Mass Transfer Intensification in the Process of Ammonia-Based Flue Gas Desulfurization. *Ind. Eng. Chem. Res.* 2020, 59, 19781-19792. DOI: https://dx.doi.org/10.1021/acs.iecr.0c04164.
